# Correlations between Psoriasis Area and Severity Index (PASI), body mass index (BMI), smoking, and alcohol consumption in patients with psoriasis – a Romanian study

**DOI:** 10.25122/jml-2026-0013

**Published:** 2026-02

**Authors:** Oana-Georgiana Văduva, Argyrios Periferakis, Lamprini Troumpata, Aristodemos-Theodoros Periferakis, Priscila Mădălina Ologeanu, Roxana Elena Doncu, Vlad Mihai Voiculescu, Călin Giurcăneanu

**Affiliations:** 1 Faculty of Medicine, Carol Davila University of Medicine and Pharmacy, Bucharest, Romania; 2 Akadimia of Ancient Greek and Traditional Chinese Medicine, Athens, Greece; 3 Elkyda, Research & Education Centre of Charismatheia, Athens, Greece; 4 Department of Gastroenterology, Emergency University Hospital, Bucharest, Romania; 5 Dermatology Department, Elias Emergency University Hospital, Bucharest, Romania

**Keywords:** psoriasis, PASI score, BMI, alcohol, smoking, diabetes

## Abstract

Psoriasis, one of the most prevalent dermatological diseases, is a chronic inflammatory condition influenced by genetic and environmental factors, such as lifestyle and nutrition. Regarding lifestyle, body weight, smoking, and alcohol consumption have been studied in the last decades, for their association with the risk of developing psoriasis and with its severity. Moreover, the association between diabetes mellitus and psoriasis severity is also under research. In our observational cross-sectional study, we examined a convenience sample of 282 patients with psoriasis vulgaris, aged 18 to 79 years. The Psoriasis Area and Severity Index (PASI) score ranged from 1 to 54, with a mean of 8.34 ± 6.69; patient body mass index (BMI) ranged from 18.80 to 57.19, with a mean of 25.96 ± 5.21, and increased with patient age. A direct correlation was observed between BMI and psoriasis severity; non-smokers generally had lower PASI scores, although the association was not statistically significant (*P* = 0.944). Similarly, PASI scores were generally higher in alcohol consumers, but this association was also not statistically significant (*P* = 0.983). A non-statistically significant increase in PASI scores was also observed in patients with diabetes as a comorbidity. Based on our study conducted on a convenience sample from a Romanian hospital, it appears that higher BMI, alcohol consumption, and smoking are associated with increased PASI score values. However, further research is needed to understand the underlying mechanisms better.

## Introduction

Psoriasis is among the most well-known inflammatory skin pathologies, predominantly presenting with cutaneous symptoms, although it may develop numerous systemic manifestations [1,2]. Even though there are significant regional variations in the incidence and prevalence of psoriasis, it is believed to affect over 100 million people worldwide [3], with people of Caucasian ancestry and those from Western countries being at higher risk of manifesting the disease [4]. Potentially, the burden of disease is even higher when considering the underdiagnosis of related comorbidities such as psoriatic arthritis [5]. Being a predominantly inflammatory pathology, the numerous associated comorbidities [6,7] negatively affect the patients’ quality of life [8].

It is well established that many patients with psoriasis have a genetic predisposition, with the genes most strongly implicated belonging to the human leukocyte antigen (HLA) complex [9]. On the other hand, not all patients with the relevant genetic background, as detailed in Dand *et al*. [10], will develop the disease. Therefore, it is considered that at least some extrinsic factors influence both the likelihood of developing the disease and, potentially, its severity [11]. Regarding these extrinsic factors, several are currently under investigation for their influence on psoriasis and their possible association with therapeutic approaches. Psychological and physiological stress are among the factors currently being studied [12]. In everyday medical practice, the severity of psoriasis in patients with a confirmed diagnosis [13] is evaluated using the Psoriasis Area and Severity Index (PASI) score, first introduced in 1978 [14].

Based on previous research efforts on the interplay between genetic and environmental factors in autoimmune diseases, particularly in patients with psoriasis [15,16], we chose to investigate the association between psoriasis severity, as quantified by the PASI score, and body mass index (BMI), smoking, and alcohol consumption habits in a convenience sample. Furthermore, evidence suggests an association between psoriasis incidence and diabetes mellitus [17,18]; based on these findings, we also examined the relationship between PASI score values and diabetes mellitus in our sample.

## Material and Methods

### Study design

This was an observational cross-sectional study including 282 patients with psoriasis vulgaris, selected between January 2022 and December 2024 at the Elias University Hospital, Bucharest, who met specified inclusion/exclusion criteria (Table 1). The sample consisted of 51.77% women (*n* = 146) and 48.23% men (*n* = 136). The inclusion criteria were patients aged 18 years or older with a confirmed diagnosis of psoriasis based on clinical criteria and histological confirmation. Patients with malignancy or infections were excluded. Patient age ranged from 18 to 79 years, with a mean age of 48.35 ± 14.04 years and a median of 49 years; no significant differences were observed between sexes.

**Table 1:** Detailed presentation of the inclusion and exclusion criteria used in our study

Inclusion criteria	Exclusion criteria
1. Age > 18 years old	1. Age < 18 years old
2. Certain diagnosis of plaque psoriasis based on clinical criteria	2. Patients with a certain diagnosis but with any concurrent malignancy
3. Certain diagnosis of plaque psoriasis based on histological criteria	3. Patients with a certain diagnosis but with a concurrent active infection

### Data collection

Collected data included demographic parameters (age, sex, residence) and lifestyle factors (smoking status, alcohol consumption), which were recorded using a structured questionnaire. The positive diagnosis of diabetes mellitus was based on the internationally accepted criteria [19].

### Statistical analysis

Statistical analyses were performed using IBM SPSS Statistics 27. Continuous variables were reported as mean ± standard deviation (SD) or median, and categorical variables were reported as frequencies and percentages. The normality of the distributions was assessed using the Kolmogorov–Smirnov and Shapiro–Wilk tests, as well as graphical analysis.

Associations between continuous variables were evaluated using the Spearman correlation coefficient. Group comparisons were conducted using Mann–Whitney U or Kruskal–Wallis tests, with Bonferroni correction applied as necessary. Effect sizes were estimated using r for Mann–Whitney tests and η^2^ for Kruskal–Wallis tests, interpreted according to Cohen’s thresholds (small, medium, large). Associations between categorical variables were analyzed using the Chi–square test, with effect size estimated via the Phi coefficient; linear-by-linear analysis was used to evaluate trends. For multivariate regression analysis, model assumptions were assessed through collinearity analysis, the Durbin–Watson test, and residual diagnostics. *P* values < 0.05 were considered statistically significant at the 95% confidence level.

## Results

### PASI score and psoriasis severity

In the analyzed sample, the PASI score varied between 1 and 54, with a mean value of 8.34 ± 6.69 and a median of 6. The mean PASI score was slightly higher in men compared with women (men: 8.66 ± 6.31; women: 8.05 ± 7.03). According to the updated diagnostic and treatment guidelines for psoriasis, PASI > 10 indicates severe psoriasis [12].

In the analyzed sample, 26.60% of the patients had severe psoriasis (PASI > 10) (*n* = 75), while 73.40% had mild to moderate psoriasis (*n* = 207).

### Patient BMI and correlation with age and PASI score

The BMI of the selected patients ranged from 18.80 to 57.19, with a mean of 25.96 ± 5.21 and a median of 23.10. BMI values for female patients ranged from 18.80 to 49.54, with a mean of 26.45 ± 5.10 and a median of 26.97. BMI values for male patients ranged from 20.70 to 57.19, with a mean of 25.44 ± 5.29 and a median of 22.7.

Based on their BMI values, 154 patients (54.61%) were within the normal limits, 85 patients (30.14%) were overweight, and 43 patients (15.25%) were obese (27 with grade I obesity, 10 with grade II obesity, and 6 with morbid obesity).

**Figure 1:**
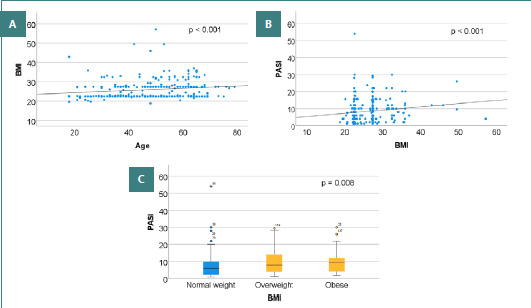
Distribution of patients according to age, BMI, and PASI scores. (A) distribution of patients according to recorded age and BMI values; (B) distribution of patients according to BMI and PASI scores; (C) distribution according to weight category and PASI scores

The Spearman correlation coefficient indicated a weak, direct association between age and BMI (rs = 0.206, *P* < 0.001), indicating that older age was associated with a significantly increased BMI (Figure 1A).

Moreover, a weak but significant correlation was observed between BMI and psoriasis severity, as assessed by the PASI score (Figure 1B), with a Spearman correlation coefficient of rs = 0.247 (*P* < 0.001). This correlation was valid across patient BMI categories; i.e., even in normal-weight patients, the higher the BMI, the more severe the form.

An increase in PASI score was observed among patients classified into higher excess body weight categories. Specifically, normal-weight patients had PASI scores ranging from 1 to 54, with a mean of 7.43 ± 6.62 and a median of 6. Overweight patients had PASI scores ranging from 1.19 to 29.40, with a mean of 9.17 ± 6.61 and a median of 8. Obese patients had PASI scores ranging from 2 to 30, with a mean of 9.96 ± 6.69 and a median of 9.6. More specifically, the mean PASI score was 9.58 ± 6.72 in patients with grade I obesity, 9.65 ± 6.65 in those with grade II obesity, and 12.18 ± 7.37 in those with morbid obesity (Figure 1C).

The Kruskal–Wallis test indicated significant differences in PASI scores among weight categories based on BMI (normal weight/overweight/obese) (χ^2^ = 9.710, *P* = 0.008, η^2^ = 0.028), revealing a small effect size according to conventional thresholds. Post-hoc analyses with Bonferroni correction showed that overweight and obese patients had significantly higher PASI scores than normal-weight patients (*P* = 0.024 and *P* = 0.007, respectively). In contrast, the difference between overweight and obese patients was not statistically significant (*P* = 0.389).

**Figure 2:**
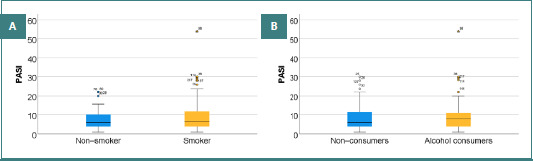
(A) Distribution of PASI scores in smokers and non-smokers; (B) Distribution of PASI scores based on the patient’s alcohol consumption.

Based on their BMI values, the patients were divided into two categories: those with normal weight and those with excess weight (overweight and all grades of obesity). An increase in PASI score was observed among patients with excess weight. Specifically, normal-weight patients had PASI scores ranging from 1 to 54, with a mean of 7.43 ± 6.62 and a median of 6. In contrast, overweight patients had PASI scores ranging from 1.19 to 30, with a mean of 9.44 ± 6.62 and a median of 8 (Figure 2A).

The Mann–Whitney U test indicated that overweight patients had significantly higher PASI scores than normal-weight patients (U = 11887.5, Z = 2.995, *P* = 0.003, r = 0.18), suggesting a small effect size.

### PASI score and smoking

The prevalence of smoking within the analyzed sample was high, with 78.01% of the patients declaring themselves to be smokers (*n* = 220). The Chi-square test indicated a significant association between the patient’s sex and smoking, while the Phi coefficient revealed a weak relationship between the two variables, χ^2^ = 11.727, ф = 0.204, p < 0.001. The smoking rate was significantly higher in men (86.76%, *n* = 118) than in women (69.86%, *n* = 102).

Similarly, the application of the Mann–Whitney U test indicated that smokers were significantly younger (47.08 ± 13.68 years) than non-smokers (52.87 ± 14.48 years) (U = 5120.5, Z = 2.997, *P* = 0.003, r = 0.18), indicating a small effect size.

As seen in Figure 2A, smokers had PASI scores ranging from 1 to 54, with a mean of 8.42 ± 6.96 and a median of 6.75. Non-smokers had PASI scores ranging from 1 to 22, with a mean of 8.07 ± 5.68 and a median of 6. The PASI score was slightly higher in smokers than in non-smokers, although the difference was not statistically significant (*P* = 0.944). 25.91% of smokers (*n* = 57), respectively 29.03% of non-smokers (*n* = 18) had severe psoriasis (PASI > 10). The difference between groups was not statistically significant (*P* = 0.628).

### PASI score and alcohol consumption

The prevalence of alcohol consumption within the analyzed sample was high, with 49.29% of the patients declaring themselves to be alcohol consumers (*n* = 139). The Chi-square test indicated a statistically significant association between the patient’s sex and alcohol consumption. At the same time, the Phi coefficient indicated a moderate correlation between the two variables, χ^2^ = 61.745, ф = 0.468, *P* < 0.001. The proportion of alcohol consumers was significantly higher among men (73.53%, *n* = 100) compared with women (26.71%, *n* = 39). The analysis did not indicate significant differences regarding the age difference between alcohol consumers and non-consumers (*P* = 0.962).

As seen in Figure 2B, alcohol consumers had PASI scores between 1 and 54, with a mean value of 8.40 ± 7.08 and a median value of 8. Non-alcohol consumers had PASI scores ranging from 1 to 30, with a mean of 8.29 ± 6.31 and a median of 6. The PASI score was slightly more elevated in alcohol consumers compared with non-alcohol consumers, even though it was not statistically significant (*P* = 0.983). 25.18% of alcohol consumers (*n* = 35), respectively 27.97% of non-alcohol consumers (*n* = 40) had severe psoriasis (PASI > 10), the difference between the groups not being statistically significant (*P* = 0.686).

### PASI score and diabetes mellitus

In the analyzed sample, 32.62% of the patients were diagnosed with diabetes (*n* = 92). The Mann–Whitney U test indicated that diabetic patients were significantly older than non-diabetic patients (U = 12,510, Z = 5.874, *P* < 0.001).

Patients with diabetes were aged between 24 and 79 years, with a mean age of 55.48 ± 11.05 years and a median age of 58 years. Non-diabetic patients were aged between 18 and 75 years, with a mean age of 44.91 ± 14.06 years and a median age of 45 years. The proportion of patients with diabetes was similar in both sexes.

**Figure 3:**
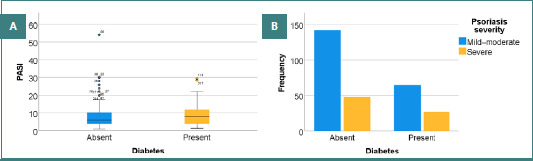
Distribution of PASI scores according to diabetes status. (A) distribution of PASI scores in patients with and without diabetes; (B) frequency of mild–moderate and severe psoriasis according to diabetes status.

As shown in Figure 3A, patients with diabetes had PASI scores ranging from 1.19 to 29.40, with a mean of 8.87 ± 6.13 and a median of 8. Non-diabetic patients had PASI scores ranging from 1 to 54, with a mean of 8.09 ± 6.94 and a median of 6. The PASI score increased slightly more in patients with diabetes than in non-diabetic patients, although the difference was not statistically significant (*P* > 0.05). 29.35% of patients with diabetes (*n* = 27) and 25.26% of non-diabetic patients (*n* = 48) had severe psoriasis (PASI > 10); the difference between the groups was not statistically significant (*P* = 0.467) (Figure 3B).

### Multivariable linear regression analysis of PASI score

A multiple linear regression model was conducted with the PASI score as the dependent variable and BMI, age, sex, alcohol consumption, smoking status, and diabetes as independent variables. Although the model was statistically significant (F = 2.584, *P* = 0.019), it explained only 5.4% of PASI variability (R2 = 0.054, adjusted R^2^ = 0.033). No evidence of multicollinearity was detected, and residual analysis did not reveal violations of model assumptions. BMI was the only variable independently associated with PASI score in the multivariable model (B = 0.205, 95% CI, 0.067–0.343; *P* = 0.004). For each one-unit increase in BMI, the PASI score increased by approximately 0.21 points after adjustment for the other variables included in the model.

## Discussion

Psoriasis incidence appears to increase with age, which is a trend supported by multiple epidemiological analyses [20,21]. This was particularly evident in high-income countries and likely reflects cumulative exposure to a range of risk factors [4].

In our sample, patient age ranged from 18 to 79 years, with a mean of about 48 years. The mean age, falling between the 20-30 and 50-60 year-old age groups, is consistent with current evidence, indicating a bimodal distribution of peak psoriasis age-related incidence in these two age groups [22,23]. It is not exactly known why there is this incidence trend. Still, it is believed to reflect distinct effects of immunogenetic and environmental factors throughout the lifespan, although the precise mechanisms remain incompletely elucidated [4]. The PASI scores in patients reporting alcohol consumption were higher, even though the difference was not statistically significant; this is in accordance with existing literature on the adverse effects of alcohol consumption on psoriasis severity.

As expected, smoking and psoriasis severity had a positive association, even though it was not statistically significant; it has already been established that smoking is a risk factor for the development of autoimmune diseases, via a number of mechanisms [24,25]. It is important to note that smoking frequently coexists with an increased BMI, which is a recognized risk factor for certain pathologies, including psoriasis [26-29]. It is generally known that obesity is linked, either directly or indirectly, to systemic inflammation [30,31], a hallmark of psoriasis. Based on our dataset, older age was associated with a significantly higher BMI; this is consistent with research evidence showing a tendency towards weight gain in older adults [32]. While the association between obesity and mortality in this age group has not been definitively proven [33], increased body weight has been demonstrated to be associated with increased pain perception [34] and modest disease risk [35]. The increase in BMI with age, as seen in our statistical analysis and the relevant scientific literature, may partly account for the peak incidence of psoriasis in the second age group. It is noteworthy that the positive correlation between BMI and PASI score is valid across patient BMI categories. Finally, no significant association was observed between diabetes mellitus as a comorbidity and patients’ PASI scores.

Our study has several limitations; to begin with, our sample size, while relatively large, did not include a representative sample from all the regions of Romania. Moreover, our analysis did not account for a family history of psoriasis; while it is true that psoriasis may develop irrespective of a predisposing genetic background, it is conceivable that genetic predisposition would increase disease severity, especially in the presence of aggravating factors such as smoking or alcohol consumption. Both for assessing smoking habits and alcohol consumption, we used structured questionnaires, which, although an accepted and tested method for data collection, do not ensure truthful and accurate reporting by the patients, introducing a certain degree of reporting and recall bias. Regarding other potential quantitative analyses, we did not evaluate serum biomarkers of inflammation, which may have provided a more accurate overview of each patient’s inflammation status. Treatment status or the presence of other comorbidities was also not factored into our data collection and subsequent statistical analyses. On the other hand, while the PASI score is a widely used tool for assessing psoriasis severity, it has several limitations, as it is not standardized [36] and it is liable to underestimate mild and moderate psoriasis (the most prevalent forms), as well as be very similar for patients with different manifestations or under treatment [37]. Last but not least, we did not account for our patients’ dietary preferences, nor did we evaluate their perceived stress levels. For the former case, it is well-established that diet may affect both the risk for developing psoriasis and its severity [12]; for the latter, increased psychological stress may lead to psoriasis exacerbations [38].

Still, regarding the strengths of our research, sufficient statistical power was achieved to detect significant associations for key variables comprising smoking, alcohol consumption, and BMI, and support the robustness of the observed correlations. Our study is also among the very few that have examined these associations solely in a Romanian population.

Future studies on larger and more diverse populations, including biochemical profiling, would allow for a more comprehensive understanding of how these modifiable factors contribute to the modulation of psoriatic disease. Other scores for estimating psoriasis severity [39] could also be used and compared to the accuracy of PASI within the same and different populations.

## Conclusion

Psoriasis is characterized by chronic inflammation and may be associated with other comorbidities, especially in patients with genetic risk factors. Apart from genetic factors, several lifestyle factors influence disease severity, as quantified by the PASI score. In our sample, consisting solely of Romanian patients, we discovered a statistically significant association between increased PASI score values and BMI; increased PASI score values were recorded in alcohol consumers and smokers, even if the association was not statistically significant. While it may be true that such lifestyle factors influence disease severity, more research is needed to uncover the precise mechanisms underlying this effect.
